# Estimation of artificial neuron parameters that obtain a required distribution of coupled system periods in a hybrid network

**DOI:** 10.1186/1471-2202-15-S1-P64

**Published:** 2014-07-21

**Authors:** Ryan M Hooper, Ruben A Tikidzhi-Khamburyan, Carmen C Canavier, Astrid A Prinz

**Affiliations:** 1 Department of Biomedical Engineering, Georgia Tech./Emory University, Atlanta, Georgia 30332, USA; 2 Department of Cell Biology and Anatomy, Louisiana State University Health Science Center, New Orleans, Lousiana 70112, USA; 3 Neuroscience Center for Excellence, Louisiana State University Health Science Center, New Orleans, Louisiana 70112, USA; 4 Department of Biology, Emory University, Atlanta, Georgia 30322 , USA

## 

Rhythmic neuronal activity is a hallmark of many higher-level functions of the nervous system including the expression of rhythmic motor patterns and generation of behaviors such as cognition, memory, arousal, and sleep. Many rhythmic networks underlying these behaviors consist of one or more pacemaker neurons that are coupled with non-pacemaking neurons, and exhibit reliable stereotyped activity that however displays diverse levels of activity variability between networks – and sometimes those circuits that exhibit high levels of activity variability even depend upon their variability to accomplish some behavioral goal [1,2]. While it is understood that synaptic feedback from non-pacemaking neurons coupled to pacemaker neurons can limit variability of rhythmic activity [3,4], it is not yet understood how specific neuronal dynamics influence variability of the coupled network. Here we develop a theoretical approach to predict how network activity variability – as quantified by the probability distribution of observed periods – changes with altered phase response dynamics of non-pacemaking neurons, and demonstrate this theoretical approach’s predictive efficacy in hybrid networks established in the stomatogastric nervous system (STNS) of *Cancer borealis.* These hybrid networks consist of one biological pacemaker neuron (Anterior Burster/Pyloric Dilator complex [AB/PD]) and one artificial non-pacemaker neuron (designed to mimic the burst timing of the Lateral Pyloric [LP] neuron), which allows us to vary the phase response dynamics of the latter element.

Our simple theoretical approach to predict the effects of network coupling on activity variability is based on three assumptions. First, within the range of variation of AB/PD neuron’ intrinsic period, it is assumed that there is always at least one stable fixed-point in the dynamics of the coupled system. Second, it is assumed that any drifting of the AP/PD neuron’s intrinsic period is significantly slower than the duration of one intrinsic period, so that the coupled system is capable of reaching a steady-state condition. Third, it is assumed that as the period of AB/PD varies, any changes in this neuron’s phase resetting curve (PRC) are negligibly small. The first two assumptions allow us to directly map an AB/PD neuron’s intrinsic period onto the network period in the coupled system. The last assumption allows us to recalculate the map for different intrinsic periods and predict the distribution of periods in the coupled network based upon the known distribution of intrinsic periods of AP/PD neuron. Using previously developed analyses [5] and a linear t_S_-t_R_ curve for the artificial neuron we are able to qualitatively predict how slope and bias of the artificial neuron’s t_S_-t_R_ curve affect the distribution of network periods.

All experiments are performed using standard intracellular electrophysiological methods for the pyloric network of the STNS, and hybrid networks are established with the dynamic clamp.
